# P-845. HECK-Yes This is The Remix!! Ceftriaxone vs Cefepime or Carbapenems for Definitive Treatment of Low-Risk AmpC-Harboring Enterobacterales Bloodstream Infections

**DOI:** 10.1093/ofid/ofae631.1037

**Published:** 2025-01-29

**Authors:** Rachel M Kenney, Michael Veve, Anita Shallal, Robert Tibbetts, Jessica L Mulbah

**Affiliations:** Henry Ford Hospital, Detroit, Michigan; Henry Ford Health, Detroit, Michigan; Henry Ford Health, Detroit, Michigan; Henry Ford Health, Detroit, Michigan; Henry Ford Health, Detroit, Michigan

## Abstract

**Background:**

Recent literature suggests ceftriaxone as a viable treatment of low-risk AmpC-producing organisms, allowing for the preservation of AmpC-stable therapies for moderate to high-risk organisms. This study aimed to determine whether ceftriaxone is effective in patients with bloodstream infections (BSI) caused by low-risk AmpC harboring Enterobacterales compared to AmpC-stable therapies.

**Figure:**
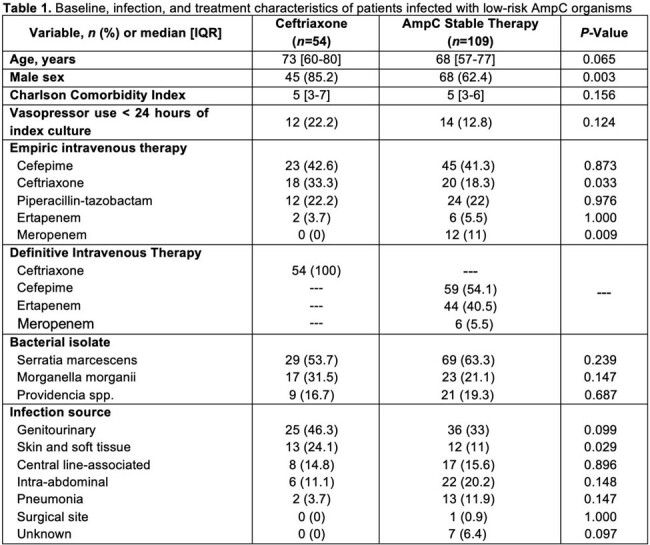

**Methods:**

This was an IRB-approved, retrospective cohort of hospitalized patients ≥18 years old with a BSI due to *Serratia marcescens*, *Morganella morganii*, or *Providencia* spp. from 1/1/2017-2/28/2024. Patients were compared according to definitive therapy with ceftriaxone vs AmpC stable therapy (cefepime or carbapenem). The primary endpoint was 30-day all-cause mortality; secondary endpoints were clinical failure, development of ceftriaxone resistance, and hospital length of stay (LOS) after index culture. Clinical failure was defined as persistent signs and symptoms of infection, repeat positive blood cultures on days 3-5 of therapy, antibiotic escalation, or death.

**Results:**

163 patients were included: 54 (33.1%) received ceftriaxone vs 109 (66.9%) AmpC stable therapies. Baseline, infection, and treatment characteristics are found in **Table 1**. 30-day all-cause mortality was observed in 5 (9.3%) ceftriaxone vs 11 (10.1%) AmpC stable patients (*P*=0.87). There were no differences in clinical success (49 [90.7%] vs 86 [78.9%], *P*=0.059), relapsing infection (3 [5.6%] vs 10 [9.3%], *P*=0.55), or rehospitalization (11 [20.4%] vs 38 [34.9%], *P*=0.06) between ceftriaxone and AmpC stable patients, respectively. Ceftriaxone resistance was only observed in AmpC stable patients (0 vs. 4 [3.7%], *P*=0.302), and median (IQR) LOS was similar between groups (5 [4-8] vs 6 [3-13] days, *P*=0.39). After adjustment for vasopressor use (adjOR 4.2; 95%CI, 1.3-13.1), ceftriaxone definitive therapy (adjOR, 0.79; 95%CI, 0.23-2.3) was not independently associated with 30-day all-cause mortality

**Conclusion:**

Patients treated with definitive ceftriaxone for low-risk AmpC Enterobacterales BSI achieved comparable outcomes to those treated with AmpC stable therapies. These findings support ceftriaxone as a treatment option for low-risk AmpC producers.

**Disclosures:**

**Rachel M. Kenney, PharmD, BCIDP**, Medtronic Inc: Spouse is an employee, stockholder

